# Risk factors and risk factor cascades for communicable disease outbreaks in complex humanitarian emergencies: a qualitative systematic review

**DOI:** 10.1136/bmjgh-2017-000647

**Published:** 2018-07-06

**Authors:** Charlotte Christiane Hammer, Julii Brainard, Paul R Hunter

**Affiliations:** Norwich Medical School, University of East Anglia Faculty of Medicine and Health Sciences, Norwich, UK

**Keywords:** systematic review, other infection, disease, disorder, or injury, public health

## Abstract

**Background:**

Communicable diseases are a major concern during complex humanitarian emergencies (CHEs). Descriptions of risk factors for outbreaks are often non-specific and not easily generalisable to similar situations. This review attempts to capture relevant evidence and explore whether it is possible to better generalise the role of risk factors and risk factor cascades these factors may form.

**Methods:**

A systematic search of the key databases and websites was conducted. Search terms included terms for CHEs (United Nations Office for the Coordination of Humanitarian Affairs definition) and terms for communicable diseases. Due to the types of evidence found, a thematic synthesis was conducted.

**Results:**

26 articles met inclusion criteria. Key risk factors include crowded conditions, forced displacement, poor quality shelter, poor water, sanitation and hygiene, lack of healthcare facilities and lack of adequate surveillance. Most identified risk factors do not relate to specific diseases, or are specific to a group of diseases such as diarrhoeal diseases and not to a particular disease within that group. Risk factors are often listed in general terms but are poorly evidenced, not contextualised and not considered with respect to interaction effects in individual publications. The high level of the inter-relatedness of risk factors became evident, demonstrating risk factor cascades that are triggered by individual risk factors or clusters of risk factors.

**Conclusions:**

CHEs pose a significant threat to public health. More rigorous research on the risk of disease outbreaks in CHEs is needed, from a practitioner and from an academic point of view.

**Key questions**

**What is already known?**

- Complex humanitarian emergencies pose significant risks to human health and communicable diseases are one of the most pressing concerns during a complex humanitarian emergency.
- Complex humanitarian emergencies exacerbate many important risk factors for outbreaks of communicable diseases.

**What are the new findings?**

- While not necessarily triggering different risk factors than other emergencies, complex humanitarian emergencies trigger more risk factor cascades with interactive feedback loops and provide a conductive environment for communicable diseases.

**What do the new findings imply?**

- Humanitarian interventions need to be aware of a wide variety of possible risk factors and to identify those most likely to trigger risk factor cascades.
- While mass population displacement triggers most other risk factors in complex humanitarian emergencies, more research is also needed on entrapment crises, which become more likely with the changing nature of conflict.

## Introduction

Complex humanitarian emergencies (CHEs^1^) pose a significant threat to public health, often in settings that were already deprived before the disruptive event or events. While CHEs generally affect the health of the affected population negatively, they especially exacerbate the risk of communicable diseases including diarrhoeal diseases, acute respiratory diseases, measles, meningitis, tuberculosis, HIV, viral haemorrhagic fevers, hepatitis E, trypanosomiasis and leishmaniosis.^2 3^ Priorities that need to be addressed in a complex emergency include rapid assessment of the health status of the affected population, mass measles vaccination, implementation of water and sanitation measures, food supply and nutrition programmes, site planning, provision of shelter, non-food items and basic medical services, control and prevention of communicable diseases and potential epidemics, surveillance and alert, mobilisation of community health workers, and coordination with national and international agencies.^3^ Several of these interventions rightly target communicable diseases, as during complex emergencies up to three quarters of excess deaths are attributable to infections.^4^

While research in this field is growing, there is inadequate understanding of the risk factors associated with communicable diseases in these situations.^5^ There is a strong need for a better evidence and understanding of the risk of communicable diseases in CHEs to inform control strategies and emergency surveillance, both of which are based on risk assessments that currently lack a common risk framework. We conducted the first (to our knowledge) systematic review on risk factors for communicable diseases in complex humanitarian emergencies.

CHEs, for our purposes, are defined as crises in a region or area in which no local coping capacity can handle the situation due to a complete breakdown of state authority. The problems in complex emergencies are diverse and a multiagency international response is necessary to address the situation. They usually result from extensive inter-state or intra-state armed conflict, leading to ‘(e)xtensive loss of life, massive displacement of population, widespread damage to societies and economies’; ‘Need for large-scale, multi-faceted humanitarian assistance’; ‘Hindrance or prevention of humanitarian assistance by political and military constraints’; ‘Significant security risks for humanitarian relief workers in some areas’.^1^ Any such situation requires a multifaceted international response, usually led by the United Nations (UN). No complex emergency would be adequately addressed by the activation of only one of the humanitarian clusters. In fact, in most complex emergencies, most if not all clusters would be activated and many such emergencies will happen in situations and countries where multiple clusters are already active due to the underlying conditions with the complex emergency exacerbating these conditions beyond the scope of an ongoing UN country programme.

## Methods

The description of methods follows the Preferred Reporting Items for Systematic Reviews and Meta-Analyses statement as far as applicable to qualitative systematic reviews.^6^ No review protocol was published beforehand.

### Inclusion criteria

For this review, we had to define three terms on which we could formulate clear inclusion criteria: (1) risk factors, (2) communicable diseases and (3) CHEs.

In order to capture all risk factors and risk factor mechanisms that might not have been labelled risk factors or been mentioned as a side note, we decided to not include terms for risk factors in our search strategy. However, they were applied as an inclusion criterion. Risk factors for this purpose were anything mentioned as increasing the risk of a communicable disease outbreak happening or as a reason for an outbreak having happened or as a mechanism that promoted favourable conditions for communicable disease spread in CHEs. Only those risk factors that apply at the population or setting level were included, as this review does not focus on the individual. Risk factors were eligible for inclusion if they could plausibly apply in CHEs.

Communicable diseases were defined as infectious diseases transmissible ‘by direct contact with an affected individual or the individual’s discharges or by indirect means (as by a vector)’.^7^

Definitions for CHEs, sometimes also simply called complex emergencies, are plentiful; however, as most agencies involved in the management of this type of disaster agree on some key issues, we used the United Nations Office for the Coordination of Humanitarian Affairs (UNOCHA) definition: “(M)ultifaceted humanitarian crisis in a country, region or society where there is a total or considerable breakdown of authority resulting from internal or external conflict and which requires a multi-sectoral, international response that goes beyond the mandate or capacity of any agency and/or the ongoing United Nations country programme”.^1^ As such, emergencies such as the 2013–2015 West Africa Ebola outbreak, the Plague outbreak in Madagascar, tsunamis,^8^ tropical storms and other disasters associated with a natural hazard are not classified as CHEs under the UNOCHA definition and therefore not eligible for inclusion in this systematic review.

We only included emergencies after 1990 and publications published on or after 1 January 1994. These dates were chosen to exclude emergencies before 1990, which were mainly influenced by the Cold War and hence considerably different in their nature. The first major CHE after the end of the Cold War was Rwanda and with those dates we made sure to include research on Rwanda but exclude research on CHEs during the Cold War.

We initially included all languages, but if no one in the research team could be found who understood the language an article was published in, we would have excluded that article for practical reasons. Because all articles found were either in English, French or Spanish, no articles were excluded due to language barriers.

### Search strategy and data sources

**Figure 1:**
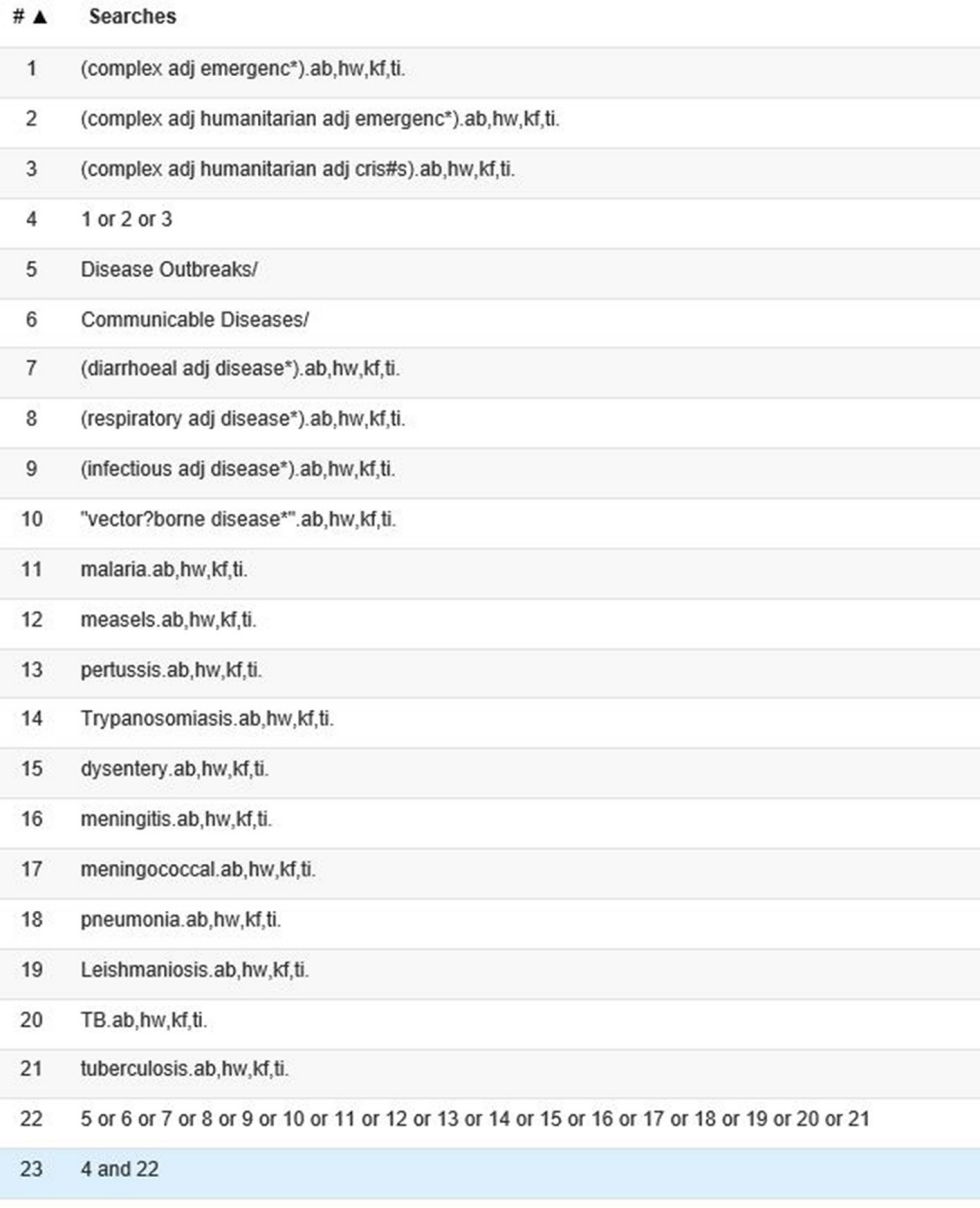
Search strategy in Medline.

Our search strategy was developed in discussion between the authors and based on previous experience and extensive background reading. The search was composed of terms for communicable diseases, including specific diseases that have very often occurred in previous CHEs and terms for CHEs. We searched the following bibliographic databases: Scopus, Medline, Embase and International Bibliography of Social Sciences (IBSS). The search strategy for Medline is presented in figure 1. Search terms for Medline and Embase included subject headings that were not available in Scopus and IBSS. The search was conducted in May 2017. Additionally, we searched the relevant websites of Medecins Sans Frontièrs, WHO and the United Nations High Commissioner for Refugees, the United Nations Children and Education Fund and ReliefWeb (UNOCHA). The search strategy was adapted for the individual websites according to the technical and search engine capacities provided by the websites. All terms were searched in abstracts and titles, keywords and relevant subject where possible. References of included publications were also checked. Reviews were included.

### Study selection

Based on the inclusion criteria, CCH and JB screened titles and abstracts of all articles identified via bibliographic databases independently. In case of disagreement, full text was obtained. An article was included for full-text review if either screener did not reject it. CCH and JB next screened full texts independently and decision about final inclusion was reached discursively. We sought access via libraries and contacted authors of conference abstracts directly.

### Data analysis and synthesis

Due to the qualitative and heterogeneous nature of the evidence found, this is a qualitative systematic review. The data were analysed using thematic synthesis.^9^ Primary coding was done by CCH, except for one article in Spanish, which was primary coded by JB. JB or CCH confirmed the primary codes and added secondary codes for all articles. Coding was done by hand and codes were transcribed into custom-made coding sheets, recording quotes, codes and subcodes. Based on the codes and subcodes, descriptive and analytical themes were developed.

## Results and discussion

**Figure 2:**
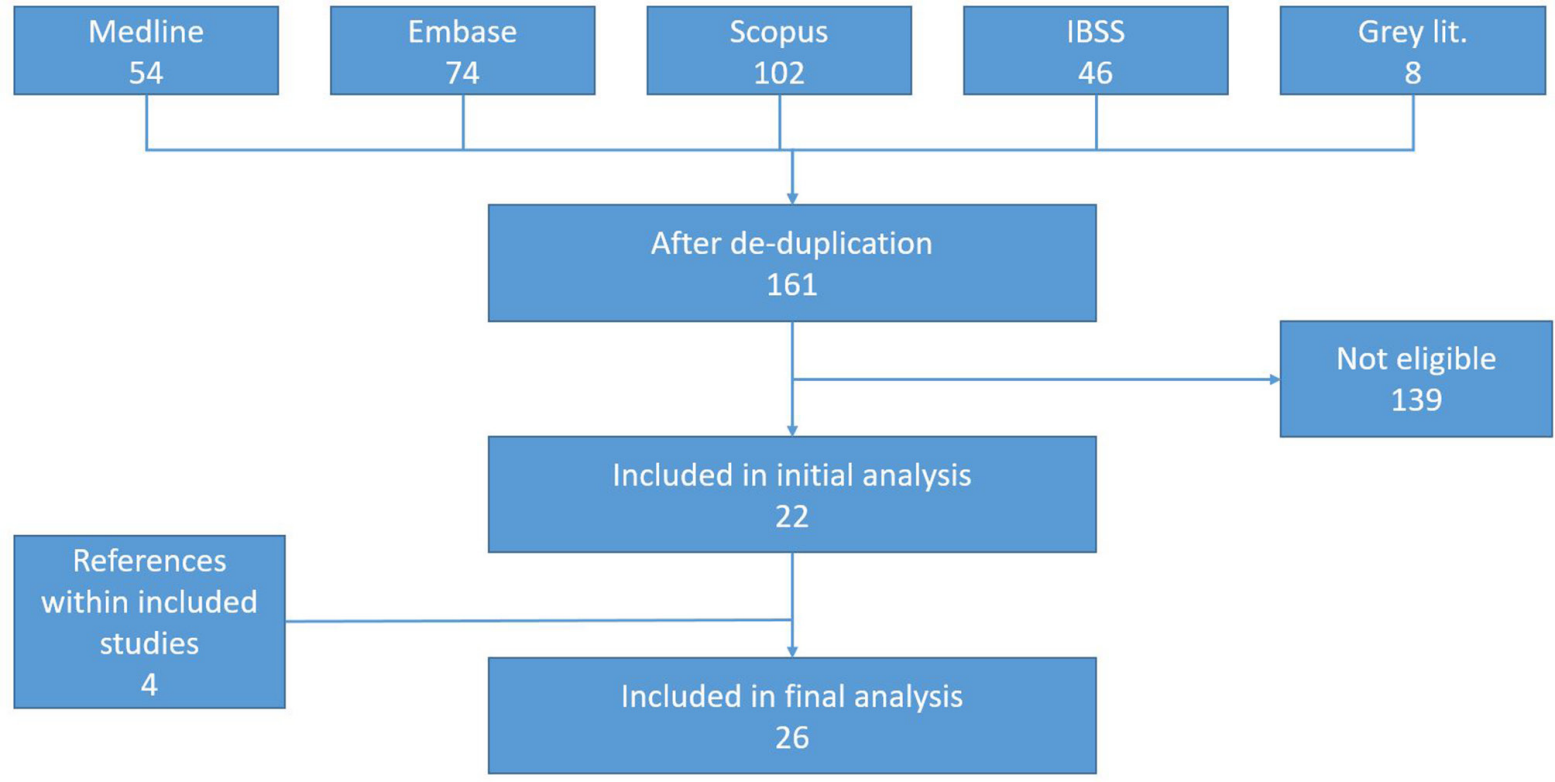
Preferred Reporting Items for Systematic Reviews and Meta-Analyses diagram. IBSS, International Bibliography of Social Sciences.

Our literature search retrieved 153 articles after de-duplication and eight grey-literature documents (as shown in figure 2). Articles were mainly excluded if they did not focus on CHEs or applied a significantly different definition of CHEs than this review does, if they did not focus on communicable diseases and if they gave no indications of any risk factors. Twenty-two articles were included directly from searches with an additional four articles retrieved from the reference lists of included articles. Articles were predominantly in English. One article was in Spanish and one in French.

Twelve main clusters of risk factors were identified that all exhibit a high level of inter-relatedness, feedback loops and interaction on various levels. These risk factor clusters provide an analytical lens and many individual risk factors can be grouped into primary and secondary (and sometimes even tertiary) clusters. Table 1 gives an overview of the included articles, the setting they describe and the risk factor clusters identified in them.

**Table 1:** List of articles included in the analysis

Article	Setting	Risk factor clusters
Abubakar *et al* 22	South Sudan; Internally Displaced Persons (IDPs) camps	Infrastructure, economy, mass population displacement, nutrition, overcrowding, water, sanitation and hygiene (WASH)
Bompangue *et al* 26	Democratic Republic of Congo; mainly refugee camps	Humanitarian response, mass population displacement
Brennan and Nandy^10^	Complex emergencies	Health and public health services, HIV-specific risk factors, humanitarian response, insecurity, mass population displacement, nutrition, overcrowding, WASH
Burkle^18^	Complex emergencies	Infrastructure, mass population displacement, overcrowding, living conditions, WASH
Burkle^24^	Complex emergencies; paediatric populations	Economy, health and public health services, mass population displacement, nutrition, overcrowding, WASH
Chaignat and Monti^12^	Complex emergencies	Environment, health and public health services, humanitarian response, living conditions, mass displacement, nutrition, WASH
Close *et al* 13	Complex emergencies	Nutrition, overcrowding, mass population displacement, health and public health services, WASH
Connolly *et al* 2	Complex emergencies	Economy, environment, health and public health services, HIV-specific risk factors, infrastructure, insecurity, mass displacement, living conditions, overcrowding, nutrition, WASH
Coulombier *et al* 14	Complex emergencies	Health and public health services, insecurity, mass population displacement, WASH
Cuadrado and Gonzalez^23^	Complex emergencies	Environment, WASH, insecurity, mass population displacement, nutrition, overcrowding, health and public health services, living conditions, economy, infrastructure
Fisher *et al* 15	Complex emergencies	Environment, health and public health services, HIV-specific risk factors, mass population displacement, overcrowding, living conditions, nutrition, WASH
Goma Epidemiology Group (1995)	Rwanda; refugee camps	Environment, WASH
Guthmann *et al* 16	Sudan; IDPs	WASH
Howard *et al* 27	Afghanistan	Economy, mass population displacement, health and public health services
Howard *et al* 25	Afghanistan	Economy, infrastructure
Khaw *et al* 28	Complex emergencies	Health and public health services, HIV-specific risk factors, insecurity, mass population displacement
Kolaczinski (2005)	Afghanistan	Health and public health services
Kolaczinski *et al* (2005)	Afghanistan	Insecurity, health and public health services
Kolaczinski and Webster (2003)	East Timor	Health and public health services, mass population displacement, overcrowding, living conditions
Leyenaar^30^	Complex emergencies	Economy, HIV-specific risk factors, insecurity, mass displacement
Liddle *et al* 31	Somalia	Economy, infrastructure, health and public health services, insecurity, mass displacement
MMWR (2011)	Horn of Africa	Mass population displacement, health and public health services
Salama and Dondero^33^	Complex emergencies	HIV-specific risk factors, insecurity, mass population displacement, health and public health services
Toole and Waldman^17^	Complex emergencies and displacement crises	Health and public health services, mass population displacement, overcrowding, living conditions, nutrition, WASH
WHO^34^	Complex emergencies	Environment, health and public health services, humanitarian response, mass population displacement, nutrition
WHO^20^	Afghanistan and neighbours	Environment, health and public health services, living conditions, mass displacement, overcrowding, nutrition, WASH
WHO^19^	Liberia	Economy, environment, health and public health services, HIV-specific risk factors, infrastructure, WASH, insecurity, living conditions, mass population displacement, overcrowding, nutrition

### Main risk factor clusters

- *WASH* 2 10–23: Water, sanitation and hygiene are central elements to limit the risk of communicable diseases in populations experiencing an emergency. As such, they are also central to CHEs and often in a more precarious state than in other emergencies. WASH risk factors include issues such as lack of safe drinking water,^2 10 12 14–17 19–21^ lack of hygiene,^10 15 19 22^ hygiene behaviour,^18 21 22^ lack of soap,^2 19–21 24^ lack of bed nets^25^ ^20^ (as vector control is usually seen as a part of WASH in humanitarian response) and general water scarcity,^2 10 12 14–17 19–21^ as well as lack of adequate sanitation and latrines. These factors considerably increase the risk for diarrhoeal diseases and compound risks for other types of communicable diseases especially if they are coupled with other risk factor categories such as overcrowding and mass population displacement.
- *Overcrowding* 2 10 13 15 17–20 22–24: Overcrowding in CHEs is usually a function of either mass population displacement or entrapment. While overcrowding can also be an issue in ad hoc shelters after the widespread destruction of homes and infrastructure, it is more prevalent if populations are forced to become refugees or internally displaced persons and are forced into camps. Overcrowding affects both hygiene-related diseases, such as diarrhoeal diseases, but also increases the transmission rate of diseases such as measles and other infections that spread from person to person.
- *Mass population displacement* 2 10 12 14 15 17–20 23 24 26–34: Mass population displacement is a trigger for most risk factor categories and as such possibly the main risk factor in CHEs. Mass population displacement is usually associated with large numbers of people moving into camp settings, often associated with overcrowding, inadequate shelter and poor WASH conditions.^2 10 15 17–20 29^ Additionally, populations are displaced into regions and areas with insufficient resources and services and with potentially increased contact of naive populations with new disease vectors. Early camp structures (such as layout of tents and siting of toileting areas) can lead to further complications. Early layout often develops as an ad hoc response to mass population displacement but may prove completely unsuitable as the camp expands.
- *Nutrition* 2 10 12 13 15 17 19 20 22–24 34: While nutrition factors such as malnutrition,^2 10 13 15 17 19 20 22 24 34^ food shortages^2 10–12 17 19^ and exposure to contaminated food^19 20^ are mainly risk factors at the individual level, they also pose increased risk to populations as a whole if a sufficient percentage of the population is exposed. Nutrition factors are related to increased susceptibility to communicable diseases with resulting greater shedding and transmission to others. At the population level, nutritional factors can exacerbate other risk factors and risk factor clusters, for example by increasing the risk of violence and social unrest. Root causes for nutrition risk factors lie mainly in other risk factor clusters such as insecurity and armed conflict or mass displacement and inadequate humanitarian response.
- *Living conditions* 2 12 19 20 23: Poor living conditions are a combination of inadequate shelter, overcrowding and other individual factors in the immediate surroundings of an individual or group of individuals. A  key risk for people uprooted from their normal lives in CHEs and subject to inadequate resources and shelter is indoor air pollution.^2 19 20^ This is due to indoor fires, both for cooking purposes and for heating.^2 19 20^
- *Insecurity* 2 10 14 19 23 28 30 31 33 35: Insecurity is a multifaceted bundle of risk factors that is one of the main root causes for increased mortality (all causes) in complex humanitarian emergencies. Insecurity is composed of factors such as armed conflict,^10^ social disruption^10 19 30 33^ and political instability.^2^ The specific nature of insecurity differs from complex emergency to complex emergency. However, by our (UNOCHA) definition, most, if not all, complex emergencies experience a high level of severe violence either from inter-state or from intra-state conflict. Insecurity triggers other factors such as a lack of an adequate humanitarian response as it poses risks to aid workers and inhibits access to beneficiaries. Additionally, it also inhibits access for the population to health services and has a high potential to disrupt all other services.
- *Infrastructure* 2 18 19 22 23 31: Due to insecurity and also in some cases long-term neglect and lack of funding, infrastructure in CHEs is often inadequate, especially in response to mass influx of people either in camps or in the community. Lack of infrastructure also often comes with a lack of domestic coordination,^2 19 31^ which additionally inhibits efficient coordination with international response. A lack of resources,^2 31^ water,^2 10 12 14–17 19–21^ electricity,^19^ funding^22^ and staff^22^ makes the affected population more dependent on an international response.
- *Humanitarian response* 10 12 26 34: By our (UNOCHA) definition, a complex emergency demands a multifaceted, multiagency international humanitarian response. However, poor response can itself become a risk for the spread of communicable diseases. Problems can lie with the response itself, due to a lack of international commitment or a lack of professionalism of the responding agencies and organisations.^12^ Problems can also arise domestically due to restrictions by governments or warring parties, unsafe conditions in which aid workers cannot properly work without unacceptable levels of risk for themselves or lack of access for various reasons.^10 34^ This also includes lack of organisational motivation^22^ and poor institutional support^10^ and complex international issues such as the lack of a binding legal framework for the protection of internally displaced populations.^24^
- *Environment* 2 12 15 19–21 23 34: Environmental factors can increase the likelihood of communicable diseases outbreaks, and this is true beyond the context of CHEs. However, many environmental factors, which would not have mattered otherwise, can be triggered by mass population displacement, especially if populations are displaced into areas with a higher prevalence of environmental risk factors. Environmental risk factors include weather and climate factors, such as cold and dust storms,^2 20^ but also vector habitats,^19 20 34^ increased contact with animals^19 20^ and endemic diseases.^2 12 19^ Mass population displacement potentially puts people at risk from these factors and also exacerbates the factors themselves due to the additional stress placed on the local environment by camps and by an influx of large numbers of people, often accompanied with significant land use changes.^19^
- *Economy* 2 19 23 25 27 30 31: While economic factors such as poverty and lack of resource are certainly issues that are important in humanitarian emergencies, they are not of the highest importance in CHEs. Poverty and economic degradation have the ability to further exacerbate the root causes of the underlying conflict but only indirectly increase the likelihood of communicable disease outbreaks.
- *Health and public health services* 2 10 12–15 17 19 20 23 24 27–29 31–36: Breakdown of health and public health services is probably one of the main risk factors for communicable diseases in CHEs both for individuals and for populations. Lack of access to health and medical care is a key risk factor for severe progressions of most communicable diseases for the individual.^2 10 12 15 17 19 20 28 29 31 33 34^ It also facilitates the further spread of communicable diseases such as tuberculosis and makes detection of cases and outbreaks harder. Additionally, in complex emergencies, public health services including vaccination, communicable disease prevention and control measures, and surveillance are no longer available making disease outbreaks more likely, harder to detect and harder to control.^2 10 12 13 15 17 19 20 24 27 31–33 35^ This breakdown of services can be seen as a function of the underlying conflict but is further compounded if there is not enough political will to provide adequate health protection.^2^
- *HIV-specific risk factors* 2 10 15 19 28 30 33: HIV is a unique and often overlooked concern in CHEs. While many of the aforementioned risk factors also apply to HIV, there are some very specific additional risk factors that are associated with an increase in the incidence of HIV in complex emergencies. Key risk factors for an increased transmission of HIV include sexual and gender-based violence,^2 10 15 19 28 30 33^ increased rates of sex work,^2 10 19 28 30 33^ use of unsafe blood products and conflict-related increased demand for (potentially unsafe) blood products,^2 19 28^ lack of infection control in healthcare facilities,^2 19 28^ lack of condoms^2 28^ and an increased use of illicit drugs.^19 28 33^ A high sexually transmitted infection prevalence can be linked to an increased risk of contracting HIV.^15^ Lack of healthcare access and lack of antiretroviral therapy increase the likelihood of vertical transmission,^30^ and mass population displacement can lead to increased contact (sexual and otherwise) with populations with a higher prevalence.^10 28 33^

### Risk factor cascades

**Figure 3:**
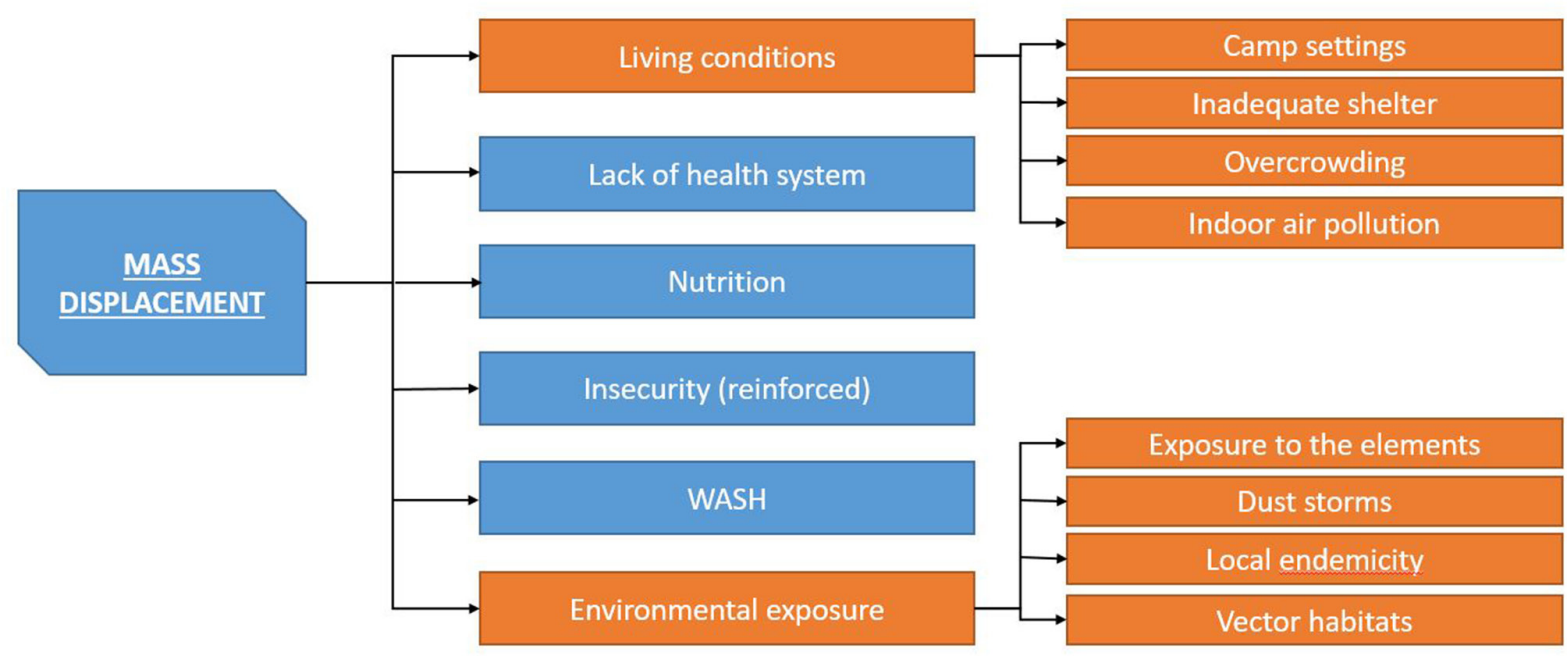
Mass population displacement cascade. WASH, water, sanitation and hygiene.

**Figure 4:**
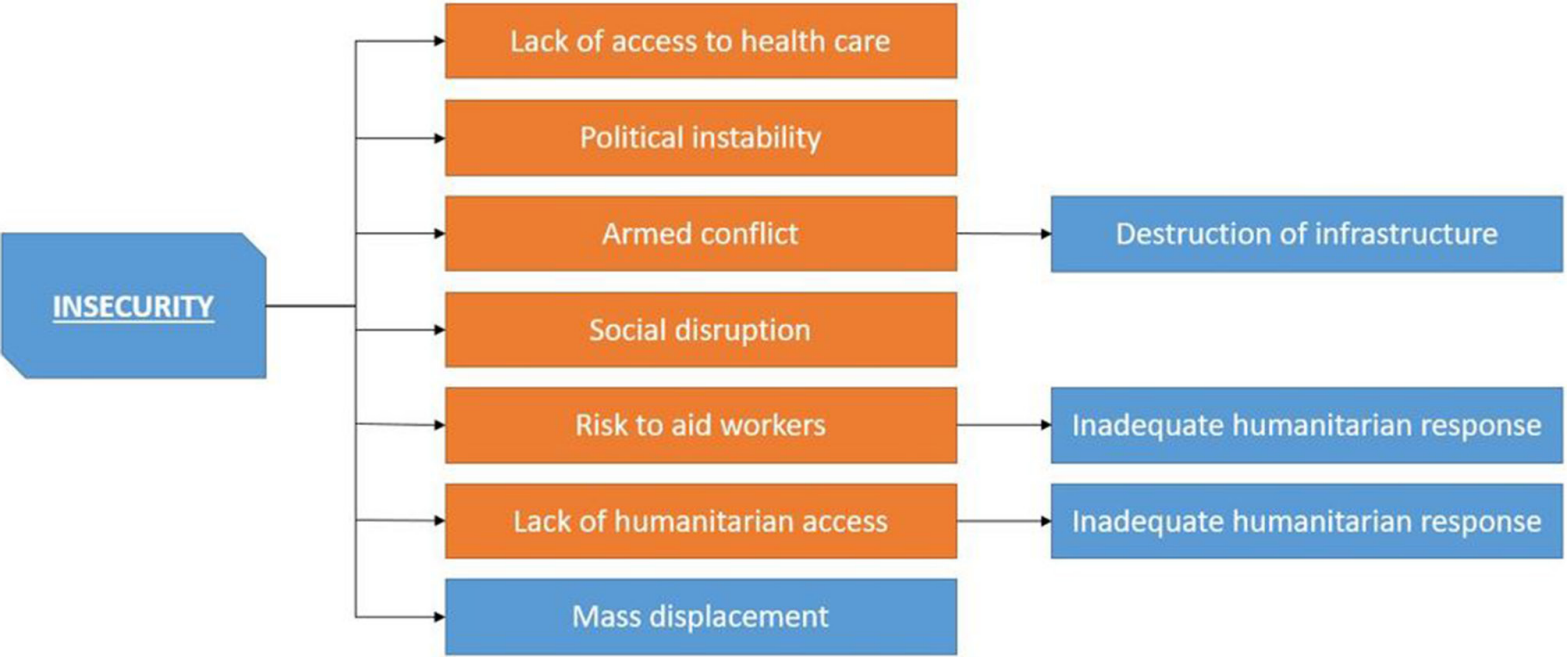
Insecurity cascade.

The risk factor clusters as well as individual risk factors often interact and exacerbate one another. Some risk factors and risk factor cluster are particularly likely to start risk cascades, especially mass population displacement (as illustrated in figure 3) and insecurity (as illustrated in figure 4).

One of the key mechanisms for driving risk factors for communicable diseases in complex emergencies is mass displacement (as shown in figure 3), especially mass displacement into camp settings.^18–20^ Camp settings enforce a high dependence on outside support for the residents. This makes residents more at risk for other risk factors. Mass displacement can reduce access to healthcare and even if access to healthcare is maintained the level and quality might be poor.^2 10 15 17 18^ Mass displacement thus tends to trigger all risk factors associated with lack of access to healthcare and increases the risks for communicable diseases both at individual and community levels. This is often coupled with living conditions that are conducive to increased transmission of communicable diseases and put the individual more at risk.^2 12 19 20^ This includes the lack of adequate shelter, which is especially prone to increase vector-borne diseases and respiratory diseases, especially in areas with cold temperatures.^2 10 15 17 19 20 24 29^ Overcrowding—often together with inadequate shelter and lack of sufficient WASH—increases the likelihood of triggering hygiene risk factors and also the transmission rate of respiratory infections and diseases such as measles. For respiratory infections, this is further exacerbated by conditions that lead to the use of indoor fires and subsequent indoor air pollution.^2 19 20^

Additionally, as human populations become more overcrowded, transmission of infections becomes more efficient, that is, the reproductive ratio (R_0_) of the infection increases.^37^ As R_0_ increases, the threshold immunisation coverage needed to achieve herd immunity also increases.^38^ Consequently, immunisation coverage that was previously sufficient is inadequate to prevent outbreaks. One of the main problems, especially in overcrowded camps, is the provision of safe water and adequate hygiene. If WASH conditions deteriorate, especially diarrhoeal disease risk increases considerably. Any insufficiency in WASH is more pronounced when coupled with high population density, as experienced in camp situations. However, mass displacement, even when not coupled with displacement into camps, also triggers additional risk factors. Displacement can be into areas with endemic diseases to which the displaced population has no immunity.^12^ Additionally, mass displacement makes populations vulnerable to environmental factors as well as reinforcing these.^12 21^ Mass displacement can exacerbate insecurity and therefore reignite a vicious circle leading to further displacement and breakdown of healthcare, services and infrastructure.

Insecurity itself, whether exacerbated by mass displacement or not, is an important triggering mechanism for communicable disease risk factors in CHEs (as shown in figure 4). Insecurity, including political instability, armed conflict and social disruption, destroys services that previously prevented the spread of communicable diseases or disallows access to these services by making accessing them unsafe.^2 10 14 19 28 30 31 33 36 39 40^ This is particularly important for healthcare services that in the last few years have increasingly become a target of armed conflict and attacks, decreasing the safety of both staff and patients.^41–43^ Additionally, disease prevention programmes are likely to be disrupted and infrastructure to be destroyed.^15 17 20 36^ With regard to humanitarian response, which can under certain circumstance step into the place of previously government-provided services, insecurity makes an adequate humanitarian response difficult.^10 34^ Not only will access to affected populations be difficult, especially in situations when insecurity and active fighting lead to entrapment or even to siege situation, as recently seen in Syria and Iraq, but insecurity also poses risks to aid workers’ security both for domestic/national and international/expatriate staff.^10 34^ Aid organisations are—understandably—increasingly reluctant to accept very high risks to their personnel, leading to gaps in provision of services, which would otherwise have been filled by a humanitarian response. Insecurity also increases the risk of the loss of domestic experts in disease prevention due to injury, death and flight.^42^

These are only some aspects of two of the many mechanisms by which CHEs drive risks for communicable diseases. We identified further cascades triggered by economics and infrastructure and risk factor cluster interaction for WASH and health systems risk factors. However, the level of complexity in these types of emergencies makes it impossible to capture all levels of interaction adequately. It is not so much that complex emergencies create different risk factors than other humanitarian crises but that they exacerbate any individual risk factors and compound interaction effects. Levels of risk factors will invariably be higher in a complex emergency and the amount of interacting risk factors creates a ‘perfect storm’^44^ where a multifaceted, well-funded and logistically and politically highly integrated humanitarian response is not possible due to political, financial or security reasons. These conditions make the danger of one or more outbreaks of communicable diseases extremely high.

While complex humanitarian emergencies do not trigger risk factors that are unknown in other types of emergencies and disasters, they produce much higher levels of risk and often tend to trigger more of the known risk factors as well as risk factor cascades. Risk factors related to poor sanitation and hygiene,^45–52^ nutrition,^46 53–55^ mass population displacement and overcrowding^47 53 56–60^ have been discussed extensively in the academic literature as being important in most types of emergencies, while risk factors resulting from an inadequate humanitarian response, armed conflict and a breakdown in government services are generally more associated with complex emergencies and other situations linked to failing statehood, such as civil war.

The question remains of how to make useful this information on risk factors and their interactions. While many of the risk factors and even starting points of risk factor cascades are addressable, the context of a complex emergency often prevents any such interventions. A key first step in any attempt to address these issues in a given complex emergency is a rapid but thorough initial needs assessment,^3 61–63^ including an assessment of the most critical risk factors present in that specific complex emergency in order to develop an evidence-based intervention strategy. However, it is unclear how to best undertake such a needs assessment. Moreover, beyond the development of evidence-based risk assessment and management methods, there is a need for more rigorous research into the operational and structural barriers that make it difficult to address risk factors in CHEs.

### Limitations

This systematic review included subjective interpretation as risk factors were rarely the main focus of the included articles. Authors do not always clearly describe the risk factors and their mechanisms. This introduced an interpretative and subjective element within the included articles, which became more subjective due to the level of interpretation required to complete the thematic synthesis. However, the authors maintained constant feedback to one another and discussed challenges, interpretations and limitations to ensure reliability and validity of the findings to the degree that a qualitative analysis allows. We are therefore confident that our interpretation properly reflects the data, although agreeing that other interpretations are possible and may be equally valid. This review was necessarily a qualitative synthesis as the evidence base (heterogeneous and qualitative in nature) did not support quantitative analysis.

## Conclusion

CHEs pose a significant threat to public health. The described cascades, interactions and feedback loops are only some of the most striking examples. The increased exposure to very many interacting risk factors and the resulting risk factor cascades created by a complex emergency encourages a perfect storm of communicable diseases risk.

However, despite these extremely increased risks and the exceptional situation that CHEs pose, we did not find a correspondingly high level of academic engagement with the issue. Most of the included articles discussed situations of mass displacement into camps, which is arguably the best studied situation concerning complex emergencies. However, conflicts like Syria and Yemen demonstrate that this might not be the most important situation in the 21st century. Syria and Yemen feature high levels of entrapment,^64–67^ as they are characterised by limited or no displacement due to a lack of safe humanitarian corridors. This situation coincides with a high level of most other risk factors, especially lack of access to healthcare, lack of humanitarian response, lack of WASH and other services, food insecurity and high levels of insecurity. We conclude that more rigorous research on the risk of communicable disease outbreaks in complex humanitarian emergencies could elucidate opportunities to either prevent or better manage such events. Such research should be undertaken in collaboration between practitioners and academics. More CHE research on entrapment situations is especially desirable, in response to the nature of recent conflicts.
